# Circulating hsa-let-7e-5p and hsa-miR-125a-5p as Possible Biomarkers in the Diagnosis of Major Depression and Bipolar Disorders

**DOI:** 10.1155/2022/3004338

**Published:** 2022-02-07

**Authors:** Dovydas Gecys, Kristina Dambrauskiene, Sandrita Simonyte, Vaiva Patamsyte, Alvita Vilkeviciute, Algirdas Musneckis, Kristina Butkute-Sliuoziene, Vaiva Lesauskaite, Lukas Zemaitis, Deimante Usaite, Virginija Adomaitiene

**Affiliations:** ^1^Institute of Cardiology, Laboratory of Molecular Cardiology, Lithuanian University of Health Sciences, Sukileliu Ave., 15 Kaunas, Lithuania; ^2^Faculty of Medicine, Departamentof Psychiatry, Lithuanian University of Health Sciences, Eiveniu St. 2, Kaunas, Lithuania; ^3^Neuroscience Institute, Laboratory of Ophthalmology, Lithuanian University of Health Sciences, Eiveniu St. 4, Kaunas, Lithuania; ^4^Life Sciences Centre, Vilnius University, Sauletekio Ave. 7, Vilnius, Lithuania

## Abstract

**Background:**

Evidence shows that microRNAs (miRNAs) could play a key role in the homeostasis and development of major depressive disorder and bipolar disorder. The present study is aimed at investigating the changes in circulating miRNA expression profiles in a plasma of patients suffering from major depressive disorder (MDD) and bipolar disorder (BD) to distinguish and evaluate these molecules as biomarkers for mood disorders.

**Methods:**

A study enrolled a total of 184 subjects: 74 controls, 84 MDD patients, and 26 BD patients. Small RNA sequencing revealed 11 deregulated circulating miRNAs in MDD and BD plasma, of which expression of 5, hsa-miR-139-3p, miRNAs hsa-let-7e-5p, hsa-let-7f-5p, hsa-miR-125a-5p, and hsa-miR-483-5p, were further verified using qPCR. miRNA gene expression data was evaluated alongside the data from clinical assessment questionnaires.

**Results:**

hsa-let-7e-5p and hsa-miR-125a-5p were both confirmed upregulated: 0.75-fold and 0.25-fold, respectively, in the MDD group as well as 1.36-fold and 0.68-fold in the BD group. Receiver operating curve (ROC) analysis showed mediocre diagnostic sensitivity and specificity of both hsa-let-7e-5p and hsa-miR-125a-5p with approximate area under the curve (AOC) of 0.66. ROC analysis of combined miRNA and clinical assessment data showed that hsa-let-7e-5p and hsa-miR-125a-5p testing could improve MDD and BD diagnostic accuracy by approximately 10%.

**Conclusions:**

Circulating hsa-let-7e-5 and hsa-miR-125a-5p could serve as additional peripheral biomarkers for mood disorders; however, suicidal ideation remains the major diagnostic factor for MDD and BD.

## 1. Introduction

Major depression disorder (MDD) and bipolar disorder (BD) are chronic, severe, and highly disabling psychiatric disorders which affect many people worldwide influencing emotional, social behaviour, and physical health [1–4]. In psychiatry, BD is characterized by repetitive episodes of mania and depression while MDD is associated with depressed mood or loss of interests in daily life [5].

The need to discover novel biomarkers or therapeutic targets to treat these disorders is actual; however, their successful identification is related to the accurate differential diagnosis of MDD and BD which can become difficult due to overlapping clinical symptoms [6, 7]. BD may be characterized with depressive episodes, and it could be easily misdiagnosed with MDD; therefore, correct differentiation between these disorders is crucial for successful treatment outcomes [5].

However, the etiology of MDD and BD is still not completely understood; the data suggest that the risk of these disorders is influenced by social, psychological, and genetic factors [8]. Pathophysiology of these mood disorders could be partially explained on changes in serotonergic and glutamatergic neurotransmission, neuroimmunity and numerous biological pathways regulating neurogenesis, and neuroplasticity signalling [4, 9, 10]. Growing genetic data show that heritability ranges in MDD and BD are 30-40% [11] and 59-85%, respectively [12, 13], while 47% genetic risk factors are common for both mood disorders [14]. High heritability suggests a crucial involvement of genetic factors in pathophysiology of MDD and BD; however, to confirm this hypothesis, additional studies are necessary in this neuropsychiatric field.

Growing evidence showed that microRNAs (miRNAs) as epigenetic modulators could play a key role in the homeostasis and development of the central nervous system (CNS) [15, 16]. miRNAs are small endogenous noncoding RNAs (20-22 nucleotides in length) that, when bound to the 3′ untranslated regions (UTRs) of messenger RNAs (mRNRs), leads to transcriptional silencing trough the mRNA deadenylation and repression of protein synthesis or degradation [17]. As posttranscriptional regulators of gene expression, microRNAs have been involved in cell development, proliferation, differentiation, DNA damage repair, and other biological processes through the organism [9, 18]. The miRNAs are short in length which allows them to bind with different affinities to almost 60% of all mammalian RNA transcripts [15]. Paracrine and endocrine signalling of miRNAs enables them to influence different gene functions at various distances and to modulate a wide range of biological pathways. Besides their presence in cells, miRNAs can be found in stable, cell-free form in the bloodstream and can be detected in various body fluids including whole blood, serum, plasma, saliva, or cerebrospinal fluid (CSF) [17]. Circulating miRNAs are packed in lipid microvesicles (exosomes, apoptotic bodies, and microparticles) or with high-density lipoproteins and are resistant to RNase activity [19].

It is important to emphasize that almost half of all identified miRNAs are expressed in the human brain, and their changes have been suggested to have effect in neural and synaptic plasticity, synaptogenesis, and other basic neuronal processes [10, 20, 21]. Significant evidence shows changes in miRNA expression profiles in postmortem brains of patients suffering from BD and schizophrenia [22, 23] and victims of suicide that suffered from major depression disorder [24, 25]. The level of miRNAs was shown to be regulated by psychotropic drugs or mood stabilizers in humans [26–28], animals [29], or cell line studies [30].

It was found that changes in the miRNA profile of peripheral blood may be correlated with variations in the neuronal tissue of various CNS-related disorders and could be used as biomarkers for molecular diagnostics [31, 32]. There is also evidence that alteration in miRNA expression may be associated with psychiatric disorders, cancer, and cardiovascular diseases [33–35]. Despite growing evidence that these miRNAs could be recognized as noninvasive biomarkers for various pathological conditions, it must be noted that they should be adequate in specificity and sensitivity for effective characterization of clinical disease diagnosis [36]. It was shown that there were intrinsic and extrinsic factors such as gender, type of sample, detection method, or data normalization that might influence the measurement of circulating miRNA levels [37–39]. Besides, evidence that the use of plasma rather than serum or whole blood will allow greater reproducibility and validation of results among miRNA studies and species [40] data varies between studies [36, 41, 42]. Therefore, there is a need for further evaluation and replication of circulating miRNA studies associated with clinical phenotypes of CNS disorders.

This study is aimed at investigating the changes in plasma miRNA expression profiles of patients suffering from MDD and BD to distinguish and evaluate the role of these molecules for accurate diagnosis of these mood disorders.

## 2. Materials and Methods

### 2.1. Study Participants

This study was performed in the Psychiatry Department of the Hospital of Lithuanian University of Health Sciences located in Kaunas city. The study included all cases of hospitalized patients in the Psychiatry Department with diagnoses of BD (regardless of the phase of the disorder) or MDD (first and recurrent episode), in the time between October 2018 and December 2019. A study included individuals aged ≥18 years who signed a written informed consent. Subjects who had concomitant medical or neurological illness or anyone of intellectual disabilities were excluded from the study, and a total of 110 attempters were included in the experimental study group: 84 MDD and 26 BD patients.

The control group was comprised of 74 healthy volunteers who were accepted in the study after initial interview in which they had to fulfill criteria to have no psychiatric disorders, no suicidal ideation, no exacerbated or acute somatic disorders, and the ability to give informed consent.

All personal data was kept pseudoanonymous, allowing for the identification of repeated participation in the study by the same person. The study protocol was approved by the Regional Medical Research Ethics Committee (no. DEP-2020).

### 2.2. Clinical Assessment

A clinical assessment was carried out by a psychiatrist on the third day after the patient's admission to the Psychiatry Department. All participant's diagnoses and suicidal behaviours were evaluated according to the Mini-International Neuropsychiatric Interview (MINI) questionnaire version 6.0 [43]. Clinical diagnosis was defined according to the ICD-10-AM criteria (http://www.who.int/classifications/icd/icdonlineversions/en/). MDD patients with moderate and severe depression episodes and BD patients regardless of the phase of the disorder were included.

Suicidal risk was evaluated by answering questions in MINI 6.0 B section related to suicidal ideations and behaviour in the month prior to the interview. In general, individuals that scored 0 were not considered a suicidal; scoring between 1 and 8 points was considered low suicide risk; scoring from 9 to 16 was considered medium suicide risk; and scoring 17 and more points was associated with high suicide risk.

Additionally, all study participants completed a self-administered questionnaire that included questions on sociodemographic characteristics and the adverse childhood experiences (ACE) [44].). Answers of ACE questionnaire were grouped into 13 domains named physical abuse, emotional abuse, contact sexual abuse, alcohol and/or drug abuser in the household, incarcerated household member, someone in the family chronically depressed, mentally ill, institutionalized or suicidal, household member treated violently, one or no parents, parental separation or divorce, emotional neglect, physical neglect, bullying, community violence, and collective violence. These domains were further analyzed by using binary version (BV) and frequency version (FV) scores. The BV score was counted as follows: if the participant answered in the affirmative (whether with once, a few times, or many times) then that the answer was counted as positive. Once completed the evaluation can range from 0 to 13. When counting the FV, participant's answers had exactly matched the written question in the questionnaire table. Like in BV, the completed evaluation can range from 0 to 13. The risk of alcohol use was evaluated by using AUDIT test [45]. According to its results, the participants that scored 0 to 7 points were considered to have low risk for alcohol addiction; 8 to 15 points—medium risk; 16 to 19 points—high risk; and 20 to 40 points—likely to have alcohol addiction.

### 2.3. Blood Sample Collection and Preparation of Plasma Specimens

The venous blood samples have been collected before breakfast in vacutainer tubes containing EDTA (BD, USA) during standard venipuncture procedure. Plasma specimens were prepared within one hour of blood collection, by two-step differential centrifugation. Vacutainer tubes were centrifuged at 1900 g for 10 min. at room temperature; 1 ml of each plasma top layer was transferred to the new 1.5 ml tubes. The tubes were repeatedly centrifuged at 16 000 g for 10 min. at 4°C to completely remove cellular debris. 400 *μ*l from each tube were aliquoted to new 1.5 ml tubes and stored at -80°C.

### 2.4. Total RNA Isolation and miRNA Sequencing

Total RNA including miRNA was extracted from plasma samples using the miRNeasy Serum/plasma kit (Qiagen, USA). During the extraction procedure, a pool of 52 QIAseq miRNA QC Spike-Ins (Qiagen, USA) was added to each sample. 2 *μ*l of RNA samples were reverse transcribed using QIAseq miRNA Library QC qPCR Assay kit on Biometra TAdvanced thermal cycler (Biometra, Germany) and evaluated with the same miRNA Library QC qPCR Assay kit (Qiagen, USA) on 7900HT Fast Real-Time PCR System (Applied Biosystems, USA). As per the manufacturer's instructions the qPCR data of specific red blood cell (RBC) miR-451a and stable miR-23a expression was used to monitor the possible presence of RBC miRNA (Suppl. Figure 1).

The miRNA discovery cohort consisted of the first 105 nonhemolyzed total RNA samples which were used for next-generation sequencing. Sequencing libraries from 18 BD, 50 MD, and 37 control subjects' total RNA samples were prepared using the QIAseq miRNA Library kit (Qiagen, USA). The quality of miRNA libraries was assessed with Agilent Bioanalyzer 2100 (Agilent, USA) capillary electrophoresis system using a High-Sensitivity DNA kit (Agilent, USA). Due to low plasma miRNA input during library preparation, the formation of adapter dimers was observed in several samples. miRNA-sized libraries (approx. size 180 bp) were extracted using the E-Gel™ SizeSelect™ II Agarose Gel system (Applied Biosystems, USA) according to the manufacturer's protocol. The quality assessment step was repeated on the Agilent Bioanalyzer 2100 device (Suppl. Figure 3). The final concentrations of libraries were determined on Qubit 3.0 fluorometer (Applied Biosystems, USA) using Qubit™ dsDNA HS Assay Kit (Applied Biosystems, USA). The libraries were pooled and denatured according to the standard Illumina NextSeq Library pooling guide. Sequencing was conducted as a single-end experiment on Illumina NextSeq 550 system using NextSeq™ 500/550 High Output Kit v2.5 (75 cycles) (Illumina, USA). All procedures were performed according to the manufacturer's instructions.

### 2.5. NGS Data Analysis

Raw reads were processed as described by Juzenas and coauthors [46]. Sequencing adaptors were removed using Cutadapt [47]. Reads that were shorter than 18 bp were discarded. The remaining reads were mapped to miRBase v22 [48] using MirAligner [49], and R package isomiRs [49] was used to generate read count matrix of miRNA-arms. miRNA sequences that were expressed (read count > 5) in at least 80% of the samples were used for further analysis. Subsequently, reads were mapped to QIAseq Spike-In sequences using Bowtie2 v2.4.1 [50]. Mapped Spike-In reads were converted to transcripts-per-million (TPM). 25 Spike-In sequences with TPM < 1 in at least 50% of the samples were considered lowly expressed and thus discarded from the analysis. A correlation matrix was formed from the remainder of 27 Spike-In read data to evaluate sample-to-sample relation. Samples with *R*^2^ < 0.7 were considered technical outliers and discarded from further analysis. miRNA reads were normalized using DESeq2 [51]. Normalized reads were subjected to a pairwise comparison between the case and control groups. The miRNAs with *P* value < 0.01 and log_2_ fold change > 0.4 were considered to be significantly differentially expressed. All differentially expressed genes were considered in the analysis, and NGS data was not further adjusted for any other covariates.

### 2.6. Quantitative Real-Time PCR Assays

The miRNA validation cohort consisted of 74 controls (31 samples already used for NGS and 43 newly collected samples), 26 BD patients (18 samples already used for NGS and 8 newly collected samples), and 84 MDD patients (44 samples already used for NGS and 40 newly collected samples). The quantitative real-time PCR analysis was carried out on 7900HT Fast Real-Time PCR System (Applied Biosystems, USA) using miRCURY LNA™ SYBR Green PCR Kit (Qiagen, USA) according to the manufacturer's protocol. The following Qiagen's miRCURY miRNA Assays were used: hsa-let-7f-5p (YP00204359), hsa-let-7e-5p (YP00205711), hsa-miR-103a-3p (YP00204063), hsa-miR-125a-5p (YP00204339), hsa-miR-139-3p (YP00205661), hsa-miR-425-5p (YP00204337), hsa-miR-483-5p (YP00205693), and UniSp-100 assay from QIAseq miRNA Library QC qPCR Assay kit. Expression levels of target miRNAs were normalized using 2^-∆∆CT^ method [52] with hsa-miR-425-5p, hsa-miR-103a-3p, and UniSp-100 spike-in as recommended in the manufacturer's manual.

### 2.7. Statistical Analysis

Normality distribution of the data was determined by the Shapiro-Wilk test. Nonparametric Mann–Whitney *U* test was used to evaluate differences between the case and control groups for continuous variables. Sex distribution within groups was tested using Fisher's exact test. qPCR outliers were identified and removed using robust regression and outlier removal method (ROUT) [53] with Q10%. Age-adjusted analysis for qPCR data was performed by omitting samples in the 25^th^ and 75^th^ percentiles (the youngest and the oldest subjects) from the dataset. Age differences were repeatedly evaluated by Mann–Whitney *U* test.

Binary logistic regression was used to generate aggregated probability values for receiver operating characteristic (ROC) curves and the area under the ROC curve (AUC) analysis. Results regarded as significant when *p* < 0.05.

## 3. Results

### 3.1. Study Overview

The study comprised of 2 research cohorts as depicted in Table 1. Plasma specimens from participants of the discovery group were used for profiling of circulating plasma miRNA by NGS, while specimens from the validation group was used for proofing of NGS data (Suppl. Figure 2) In both cohorts, control subjects were significantly younger than patients. The distribution of sex within groups was the same in both cohorts.

### 3.2. NGS Data Overview

A total of 105 samples have been sequenced, resulting in 1.05 B raw reads. After an adapter and quality trimming step, 715.64 M (65%) of reads were retained of which 114.27 M (17%) were mapped to 322 known human miRNAs. None of the mapped miRNAs were unique to the BD, MDD, or control group. After filtering out miRNAs that were lowly expressed (expression level < 5) and removing samples with *R*^2^ < 0.7 sample-to-sample Spike-In correlation, the miRNA sequencing dataset consisted of 125 miRNAs expressed at detectable levels in 18 BD, 50 MD, and 37 control samples.

To identify putative circulating miRNAs as candidates for BD and MDD diagnostic biomarkers, plasma miRNA expression profiles have been evaluated. As expected for multifunctional diseases, the low variance of miRNA expression data between all samples has been observed (Suppl. Figure 4). Nevertheless, we identified 5 miRNAs in BD and 11 miRNAs in MDD (Table 2, Suppl. Figure 5) that were significantly upregulated compared to the control group. However, no significant difference has been detected between the BD and MDD groups. In accordance with our sequencing data, 5 circulating miRNAs, hsa-miR-483-5p, hsa-miR-139-3p, hsa-let-7f-5p, hsa-let-7e-5p, and hsa-miR-125a-5p, have been selected for validation by RT-qPCR.

### 3.3. Real-Time qPCR Validation

**Figure 1:**
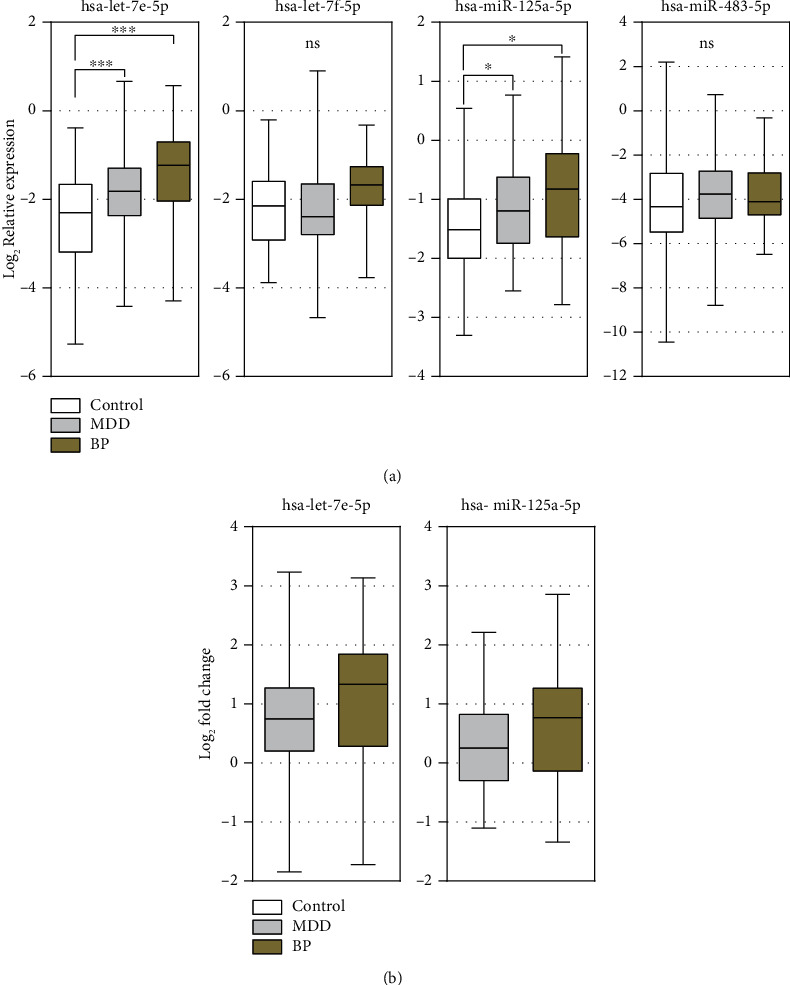
Summary of hsa-let-7e-5p, hsa-let-7f-5p, hsa-miR-125a-5p, and hsa-miR-483-5p qPCR data. (a) Relative expression data shows significant changes in hsa-let-7e-5p and hsa-miR-125a-5p expressions in plasma during BD and MDD. (b) Both hsa-let-7e-5p and hsa-miR-125a-5p were upregulated by 0.75-fold and 0.25-fold, respectively, in the MDD group as well as 1.33-fold and 0.77-fold in the BD group. Mann–Whitney *U* test was used to evaluate differences between study groups. ^∗^*p* < 0.05, ^∗∗∗^*p* < 0.001; ns: nonsignificant.

Unfortunately, it was not possible to accurately measure levels of hsa-miR-139-5p in plasma, as PCR output was characterized by several unspecific fragments; thus, hsa-miR-139-5p was not analyzed any further. Expression levels of hsa-let-7e-5p, hsa-let-7f-5p, hsa-miR-483-5p, and hsa-miR-125a-5p was determined in 184 plasma samples by qPCR. All tested miRNAs except hsa-let-7f-5p were confirmed to be upregulated in the MDD group; however, increase in hsa-miR-483-5p expression was not significant. hsa-let-7e-5p expression was significantly (*p* < 0.001) upregulated by 0.75-fold while levels of hsa-miR-125a-5p were increased by 0.25-fold (*p* < 0.05) in MDD patients' blood plasma (Figure 1). Similar results were observed after evaluating qPCR results from BD samples. All four of the tested miRNAs were found upregulated in BD patients' plasma. Nevertheless, an upturn of hsa-let-7f-5p and hsa-miR-483-5p expressions was not significant. hsa-let-7e-5p and hsa-miR-125a-5p were confirmed as significantly overexpressed with 1.33-fold (*p* < 0.001) and 0.77-fold (*p* < 0.05) higher expression in plasma than control subjects (Figure 1(b)).

**Figure 2:**
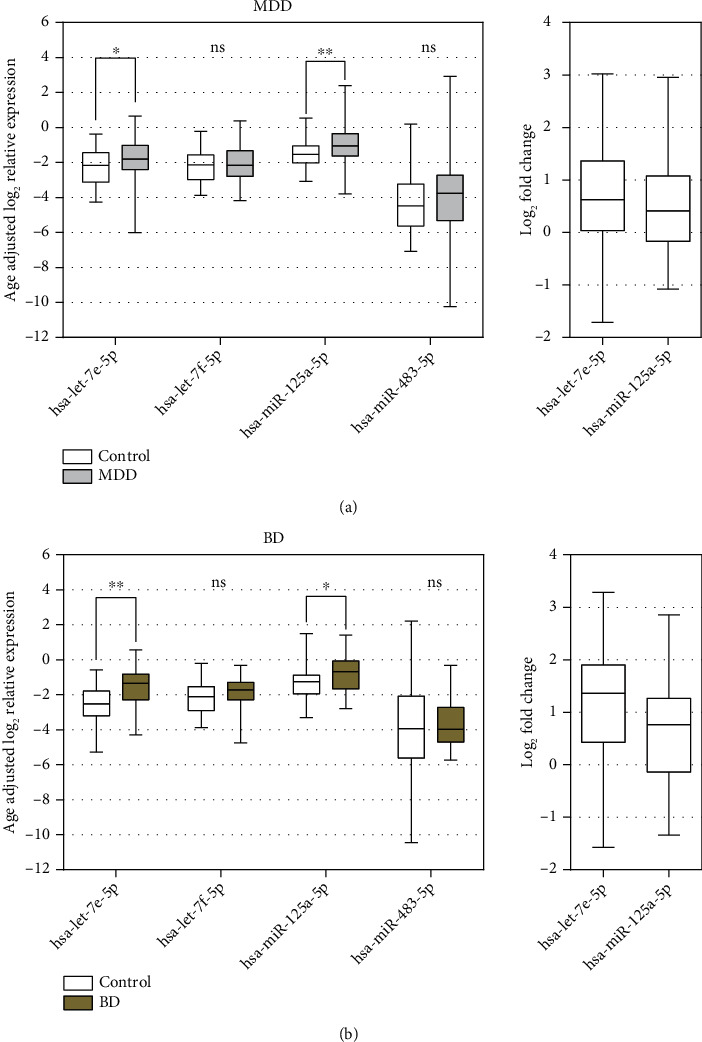
Age-adjusted relative expression analysis of hsa-let-7e-5p, hsa-let-7f-5p, hsa-miR-125a-5p, and hsa-miR-483-5p in plasma of (a) MDD and (b) BD patients. Mann–Whitney *U* test was used to evaluate differences between study groups. ^∗^*p* < 0.05, ^∗∗^*p* < 0.01; ns: nonsignificant.

Since control subjects were significantly younger than patients, hsa-let-7e-5p, hsa-let-7f-5p, hsa-miR-483-5p, and hsa-miR-125a-5p expression changes were evaluated in age-adjusted groups. Similar results were observed. Both hsa-miR-125a-5p and hsa-let-7e-5p were upregulated by 0.63-fold and 0.41-fold, respectively, in the MDD group (Figure 2(a)) as well as 1.36-fold and 0.68-fold in the BD group (Figure 2(b)).

### 3.4. Receiver Operator Curve Characteristics

hsa-let-7e-5p and hsa-miR-125a-5p expression data, as well as suicidal ideation, FV, BV, and AUDIT scores, were evaluated for diagnostic propriety. MDD and BD patients had higher median suicidal ideation and scored significantly more points in both FV and BV when compared to control subjects; however, median AUDIT scores were significantly lower in the MDD group (Table 1). To evaluate hsa-let-7e-5p and hsa-miR-125a-5p diagnostic potentials, receiver operating characteristic (ROC) analysis was performed. Both hsa-let-7e-5p and hsa-miR-125a-5p showed mediocre sensitivity and specificity in distinguishing MDD and BD patients from control subjects with area under the curve (AUC) of 0.66 and 0.6, respectively, in the MDD group. Similar results were observed in BD patients, where AUC reached 0.73 and 0.66 for hsa-let-7e-5p and hsa-miR-125a-5p, respectively (Table 3).

The same tests were performed to evaluate diagnostic sensitivity and specificity of suicidal ideation as well as FV, BV, and AUDIT scores; results are depicted in Table 3. All three questionnaire scores showed an average performance in the determination of diagnosis while suicidal ideation proved to be the most accurate indicator for subjects' condition.

**Figure 3:**
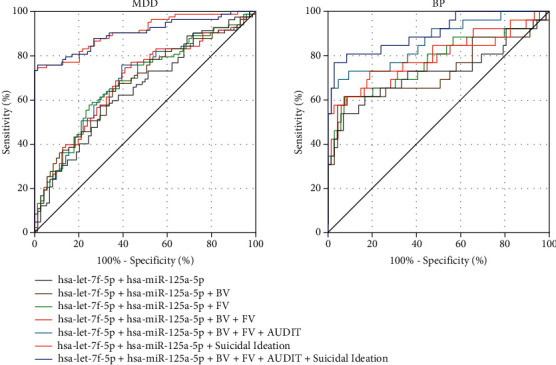
ROC analysis of aggregated miRNA and questionnaire values. ROC analysis shows that testing the hsa-let-7e-5p and hsa-miR-125a-5p expressions can improve sensitivity and specificity when making a diagnosis; however, the major determinant in subject's condition remains suicidal ideation.

To determine the impact of hsa-let-7e-5p and hsa-miR-125a-5p expression levels on diagnostic power of used questionnaires, aggregated probability values were generated using binary logistic regression with miRNA expression data and questionnaire scores as covariates. Baseline for evaluating AUC improvement using aggregated probability values were considered average scores of AUC values depicted in Table 3. Analysis showed that hsa-let-7e-5p and hsa-miR-125a-5p could improve both sensitivity and specificity of questioners by approximately 8% and 13% in the MDD and BD groups, respectively (Figure 3). Exact values are represented in Table 4.

## 4. Discussion

Due to the clinical and etiological heterogeneity of MDD and BD, it is difficult to define precise diagnosis; however, data shows that genetic and environmental stressors are involved in the pathology of these diseases [4, 8, 54]. In the past decade, multiple studies show that deregulation and dysfunction of miRNAs play important role of the pathophysiology of neuropsychiatric diseases including MDD and BD [4, 9, 10]. Changes in the peripheral miRNA expression profile of these mental disorders may be associated with changes in neural plasticity, neurogenesis, neuroimmunity, and stress response [55, 56]. When degradation or translational silencing of their mRNA targets is induced, miRNAs are represented as important epigenetic regulators of gene expression, presenting potential to become clinically applicable biomarkers when evaluating individual etiology risks of these disorders.

In the present study, using next-generation sequencing 5 upregulated miRNAS in BD and 9 upregulated miRNAs in MDD patients' blood plasma were identified. RT-PCR validation assay confirmed a significant upregulation of 2 out of 5 selected miRNA expressions: hsa-let-7e-5p and miR-125a-5p. These miRNAs have been previously associated with basic neuronal processes suggesting their important role in pathogenesis of mood disorders [4, 57, 58]. Members of the let-7 family are highly expressed in human brain and have been shown to influence neurogenesis and synapse formation [59, 60]. hsa-let-7e-5p was found downregulated in the peripheral whole blood [4] and mononuclear cells (PBMC) of depressed patients [61]. In meta-analysis, the deregulated expression of hsa-let-7e-5p has been also associated with MDD [57]. The bioinformatics analysis revealed that hsa-let-7e-5p regulates genes involved in MAP/neurotrophin/nerve growth factor receptor/Toll-like receptor 2 and 4 signalling pathways. Experimental evidence indicates that stress or depressive episode triggers innate immune response followed by increase of proinflammatory cytokines which may contribute to mental disorders pathology [62]. This data is confirmed by increased expression of innate immune genes and proteins in postmortem brain samples from depressed patients that committed suicide [63]. Moreover, it was demonstrated that hsa-let-7e-5p could be a possible target of antipsychotic drugs and mood stabilizers. In the peripheral blood of MDD patients after 12 weeks of treatment with escitalopram, hsa-let-7e-5p and hsa-let-7f-5p were found upregulated [27]. Similar results were detected in the study where the level of hsa-let-7e in peripheral blood mononuclear cells was increased after selective serotonin reuptake inhibitors (SSRIs) treatment [61].

In the current study, it was found that has-miR-125a-5p is upregulated in both MDD and BD patients' plasmas when compared to control subjects. Similar results were obtained by Wan and coauthors in a study evaluating serum of patients with depression [64] and another study conducted by Camkurt and colleagues [65]. Upregulated miR-125a-5p was also demonstrated in the frontal cortex of mice after acute stress [66]. However, Cao and colleagues have identified that miR-125a-5p is downregulated in rat hippocampus after chronic mild stress and recovers to normal level after intervention with antidepressant medication [55]. It was also suggested that has-RNA-125a-5p may regulate several target genes such as serine protein kinase (*AKT)*, serotonin receptor *(HTR2C*), corticotrophin-releasing hormone receptor *(CRHR1*), and glutamate transporter (*SCL1A2)* and mainly belong to PI3K)/Akt/neurotrophin/mammalian target of rapamycin (mTOR) signalling pathways [21].

hsa-let-7f-5p and miR-483-5p expression data obtained by NGS was not verified by qPCR; however, it would be incorrect to suggest that these miRNAs do not have a significant role in MDD and BD. This could be a result of differences between discovery and validation cohorts (e.g., medications). Previous studies have found that hsa-miR-483-5p expression correlates with insulin-like growth factor 2 (IGF2), abundantly found in the central nervous system [67]. IGF2 proteins have been associated with the phosphoinositide 3-kinase (PI3K/Akt) signalling pathway which mediates the proliferation of neural stem cells [68]. Recent data from human and animal studies show that deregulation of IGF2 may increase the susceptibility to multiple diseases, including psychiatric and neurological disorder. It was reported that administration of antidepressants upregulated IGF2 levels in the mouse hippocampus [69, 70]. It was also suggested that an effective treatment of lithium for bipolar disorder could be partially explained by reduced DNA methylation at the IGF2/H19 imprinting control region in mouse embryonic and neural stem cells [71]. Moreover, it was demonstrated that miR-483-5p regulates the level of methyl CpG-binding protein 2 (MeCP2) which is an epigenetic regulator of gene expression that is critical for normal brain function [72, 73].

There are still some inconsistencies between results of some different studies. It can be partially explained by different inclusion criteria or the technique used for the miRNA detection. Recent studies show that miRNA quality, content, and profile are associated with the origin of the sample as well. Furthermore, it is very important to point out the scientific weight of the medication used by the study participant during studies which can cause variation of miRNA expression profiles.

## 5. Limitations of the Study

Drug usage could affect miRNAs' transcriptional evaluation in the disease state. This study does not include data on lifetime exposure to antipsychotics, number of years of usage, and the type of medications. Further studies would be needed to identify if the drug usage is a significant confounding variable for gene expression perturbations. The control group was significantly younger than both MDD and BD patients in the discovery cohort and MDD patients in the validation cohort. Age is an important variable in miRNA expression levels; however, we were unable to check the impact of patient's age on identified miRNAs. Additionally, severity of the MDD and BD was not measured, which could have provided further insights in miRNA relation to mood disorders.

## 6. Conclusions

Major depression disorder and bipolar disorder are few of the most frequent and severe mental health disorders, influencing emotional and social behaviour as well as the quality of life. As these illnesses are considered multifactorial disorders, diagnostics of such conditions remain limited. This study identified that hsa-let-7e-5p and miR-125a-5p may be associated with MDD and BD. Our data shows that changes in expression of circulating miRNAs, such as hsa-let-7e-5 and hsa-miR-125a-5p, could serve as additional peripheral biomarkers for diagnosis of MDD and BD; however, further studies are necessary to identify the diagnostic potential of plasma miRNAs for mood disorders.

## Figures and Tables

**(a):** 

Discovery cohort				
	Control (*N* = 37)	MDD (*N* = 50)	BD (*N* = 18)	*p* value
Age median, years (min-max)	26 (21-65)	49 (19-75)	47 (18-69)	0.004 ^∗^ 0.006 ^∗∗^
Male, *n* (%)	8 (21.6)	12 (25.0)	7 (36.8)	0.799 ^∗^ 0.339 ^∗∗^
Female, *n* (%)	29 (78.4)	36 (75.0)	12 (63.2)	

**(b):** 

Validation cohort				
	Control (*N* = 74)	MDD (*N* = 84)	BD (*N* = 26)	*p* value
Age median, years (min-max)	46 (21-68)	52 (19-75)	47.5 (18-77)	0.008 ^∗^ 0.366 ^∗∗^
Male, *n* (%)	20 (27.0)	16 (19.0)	11 (42.31)	0.232 ^∗^ 0.147 ^∗∗^
Female, *n* (%)	54 (73.0)	68 (81.0)	15 (57.69)	
Suicidal ideation, *n* (%)	—	60 (71)	13 (50)	<0.0001 ^∗^ <0.0001 ^∗∗^
BV score median (min-max)	4 (0-12)	5.5 (0-13)	6 (0-12)	0.017 ^∗^ 0.026 ^∗∗^
FV score median (min-max)	2 (0-13)	3 (0-11)	4 (0-12)	0.0008 ^∗^ 0.0013 ^∗∗^
AUDIT score median (min-max)	3 (0-13)	1 (0-31)	1.5 (0-29)	0.011 ^∗^ 0.628 ^∗∗^

^∗^Control vs. MDD, ^∗∗^control vs. BD. Mann–Whitney *U* test was used to determine significance of age, suicidal ideation, BV, FV, and AUDIT scoring differences between study groups. Chi square test was used to evaluate sex distribution between study groups.

**Table 2:** Differentially expressed miRNAs between the BD, MDD, and control groups. Highlighted miRNAs were used for further validation experiments.

miRNA	Log_2_ fold change	*p* value
Control vs. bipolar disorder		
hsa-miR-139-3p	1.25	<0.0001
hsa-miR-483-3p	0.76	0.0022
hsa-miR-483-5p	0.61	0.0077
hsa-miR-125b-5p	0.55	0.006
hsa-let-7f-5p	0.41	0.0019
Major depressive disorder vs. bipolar disorder		
hsa-miR-423-5p	0.38	0.0014
hsa-miR-126-5p	-0.31	0.0039
hsa-miR-486-5p	0.24	0.0117
Control vs. major depressive disorder		
hsa-miR-483-5p	1.01	<0.0001
hsa-let-7f-5p	0.47	<0.0001
hsa-let-7e-5p	0.53	0.0016
hsa-miR-122-5p	0.73	0.0015
hsa-miR-125a-5p	0.42	0.0026
hsa-miR-150-5p	0.46	0.0026
hsa-miR-139-3p	0.74	0.0023
hsa-miR-193a-5p	0.60	0.0036
hsa-miR-125b-5p	0.44	0.0052
hsa-miR-197-3p	0.43	0.0087
hsa-miR-483-3p	0.45	0.0092

**Table 3:** Summary of ROC analysis of hsa-let-7e-5p, hsa-miR-125a-5p, suicidal ideation, FV, BV, and AUDIT scores. The control group was used as reference in ROC analysis.

	MDD		BD	
	AUC	*p* value	AUC	*p* value
hsa-let-7e-5p	0.66	<0.001	0.73	<0.001
hsa-miR-125a-5p	0.60	0.048	0.66	0.048
Suicidal ideation	0.85	<0.001	0.75	<0.001
FV	0.62	0.044	0.71	0.002
BV	0.61	0.018	0.65	0.028
AUDIT	0.61	0.013	0.54	0.537

**Table 4:** AUC values from aggregated ROC analysis.

	MDD		BD	
	AUC	*p* value	AUC	*p* value
FV+BV+AUDIT	0.63 (↑ 2%)	0.004	0.68 (↑ 7%)	0.012
FV+BV+AUDIT+suicidal ideation	0.87 (↑ 29%)	<0.0001	0.90 (↑ 37%)	<0.0001
hsa-let-7e-5p+hsa-miR-125a-5p	0.64 (↑ 2%)	0.0014	0.72 (↑ 4%)	0.0007
hsa-let-7e-5p+hsa-miR-125a-5p+suicidal ideation	0.91 (↑ 7%)	<0.0001	0.80 (↑ 7%)	<0.0001
hsa-let-7e-5p+hsa-miR-125a-5p+FV	0.68 (↑ 10%)	<0.0001	0.77 (↑ 17%)	<0.0001
hsa-let-7e-5p+hsa-miR-125a-5p+BV	0.67 (↑ 10%)	0.0002	0.73 (↑ 12%)	0.0006
hsa-let-7e-5p+hsa-miR-125a-5p+FV+BV	0.69 (↑ 12%)	<0.0001	0.78 (↑ 15%)	<0.0001
hsa-let-7e-5p+hsa-miR-125a-5p+FV+BV+AUDIT	0.69 (↑ 13%)	<0.0001	0.86 (↑ 36%)	<0.0001
hsa-let-7e-5p+hsa-miR-125a-5p+FV+BV+AUDIT+suicidal ideation	0.90 (↑ 3%)	<0.0001	0.91 (↑ 1%)	<0.0001

## Data Availability

Mapped sequencing reads and qPCR data will be available to researchers on request.

## Supplementary Materials

Supplementary Materials Supplementary Figure 1: representation of miR-451a and miR-23a ∆Ct data. hsa-miR-451a and hsa-miR-23a, the red blood cell-specific miRNAs, were used to evaluate hemolysis. Samples only with ∆Ct (^miR-451a–miR-23a^) lower than 8 were considered of adequate quality and were used for further experiments. Supplementary Figure 2: the design of the study. Supplementary Figure 3: electrophoreograms of miRNA library before (A) and after (B) size selection with E-Gel™ SizeSelect™ II Agarose Gel system. A miRNA-sized library is approximately 180 bp size. In some samples, large (greater than 25% of the height of the miRNA peak) peaks were observed at approximately 157 bp (adapter dimers) which is considered normal, due to extremely low total RNA input during library preparation. In order to deplete adapter dimers, cDNA libraries were size selected with E-Gel™ SizeSelect™ II Agarose Gel system (Applied Biosystems). The system uses precast 2% agarose gels with premade input and output wells, as well as an E-gel electrophoresis device with integrated power source and UV illumination. SizeSelect™ II Agarose gels provide the ability to aspirate the fragments of the desired size using automatic pipettors, thus avoiding gel excision and cDNA extraction procedures. Supplementary Figure 4: the principal components analysis of the sample groups. Low circulating miRNA expression variance has been observed within sample groups. Supplementary Figure 5: volcano plot of normalized differential miRNA expression NGS data in the BD and MDD groups. ^∗^Log_2_ fold change of 1.01 and *p* value < 0.0001.
