# Supplementary material for: Circulating hsa-let-7e-5p and hsa-miR-125a-5p as Possible Biomarkers in the Diagnosis of Major Depression and Bipolar Disorders
Source: Dis Markers. 2022 Feb 7;2022:3004338. doi: 10.1155/2022/3004338 (PMC8844308; doi:10.1155/2022/3004338)
Supplement: Supplementary Materials — Supplementary Figure 1: representation of miR-451a and miR-23a ∆Ct data. hsa-miR-451a and hsa-miR-23a, the red blood cell-specific miRNAs, were used to evaluate hemolysis. Samples only with ∆Ct (miR-451a–miR-23a) lower than 8 were considered of adequate quality and were used for further experiments. Supplementary Figure 2: the design of the study. Supplementary Figure 3: electrophoreograms of miRNA library before (A) and after (B) size selection with E-Gel™ SizeSelect™ II Agarose Gel system. A miRNA-sized library is approximately 180 bp size. In some samples, large (greater than 25% of the height of the miRNA peak) peaks were observed at approximately 157 bp (adapter dimers) which is considered normal, due to extremely low total RNA input during library preparation. In order to deplete adapter dimers, cDNA libraries were size selected with E-Gel™ SizeSelect™ II Agarose Gel system (Applied Biosystems). The system uses precast 2% agarose gels with premade input and output wells, as well as an E-gel electrophoresis device with integrated power source and UV illumination. SizeSelect™ II Agarose gels provide the ability to aspirate the fragments of the desired size using automatic pipettors, thus avoiding gel excision and cDNA extraction procedures. Supplementary Figure 4: the principal components analysis of the sample groups. Low circulating miRNA expression variance has been observed within sample groups. Supplementary Figure 5: volcano plot of normalized differential miRNA expression NGS data in the BD and MDD groups. ∗Log2 fold change of 1.01 and p value < 0.0001. [file 3004338.f1.docx]

**SUPPLEMENTARY INFORMATION**

**SUPPLEMENTARY FIGURES**

**Supplementary Figure 1.** Representation of miR-451a and miR-23a ∆Ct data. Hsa-miR-451a and hsa-miR-23a, the red blood cell specific miRNAs, were used to evaluate hemolysis. Samples only with ∆Ct (^miR-451a – miR-23a^) lower than 8 were considered of adequate quality and were used for further experiments.


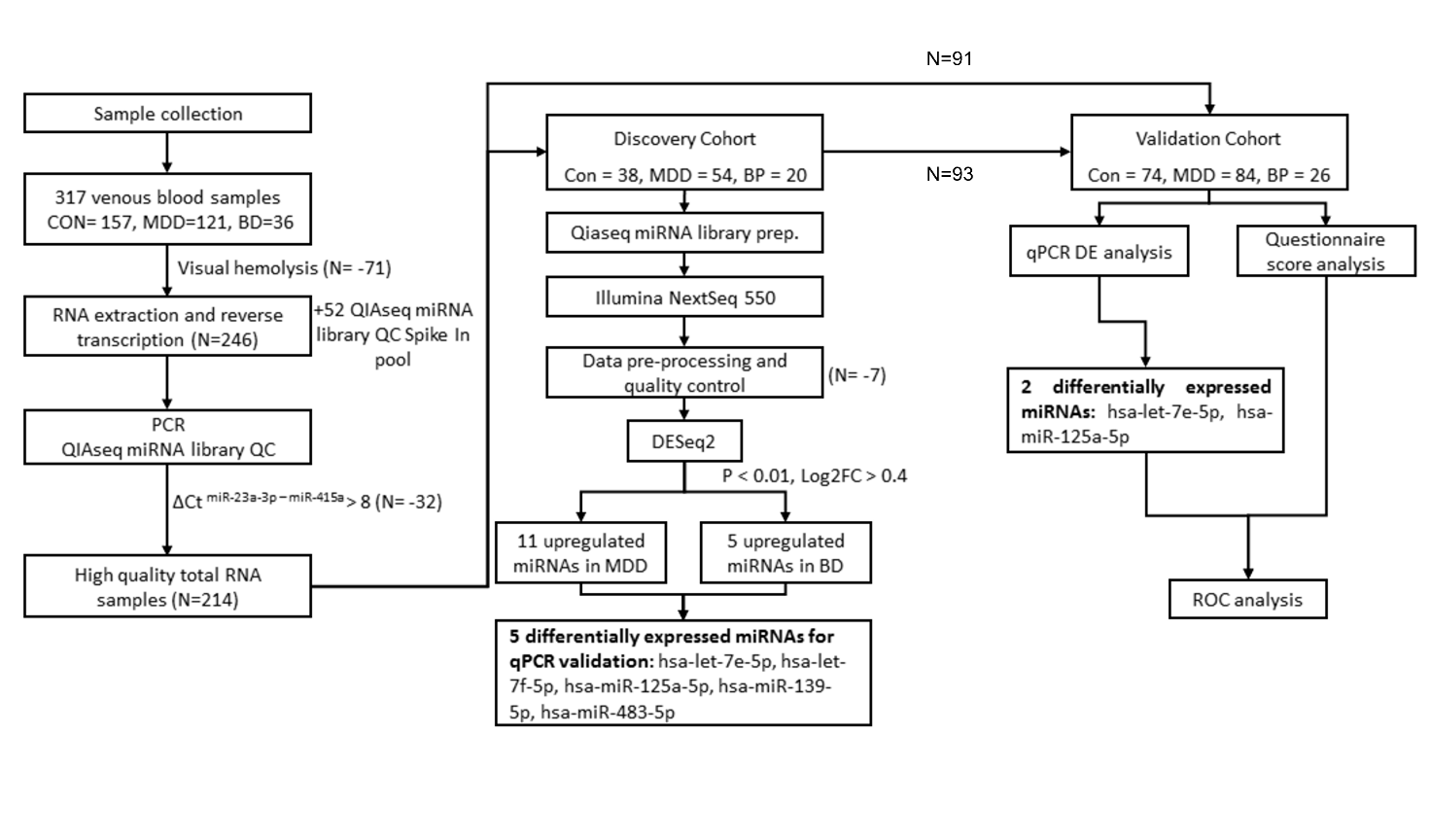


**Supplementary Figure 2**. The design of the study.


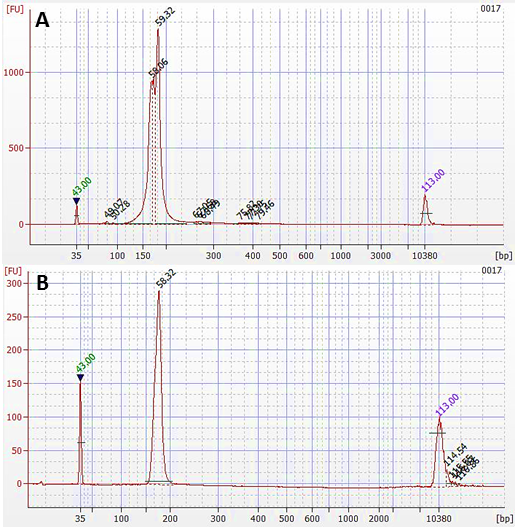


**Supplementary Figure 3**. Electrophoreograms of miRNA library before (A) and after (B) size-selection with E-Gel™ SizeSelect™ II Agarose Gel system. A miRNA-sized library is approximately 180 bp size. In some samples, a large (greater than 25% of the height of the miRNA peak) peaks were observed at approximately 157 bp (adapter dimers) which is considered normal, due to extremely low total RNA input during library preparation. In order to deplete adapter dimers, cDNA libraries were size-selected with E-Gel™ SizeSelect™ II Agarose Gel system (Applied Biosystems). The system uses pre-cast 2% agarose gels with premade input and output wells, as well as an E-gel electrophoresis device with integrated power source and UV illumination. SizeSelect™ II Agarose gels provide the ability to aspirate the fragments of the desired size using automatic pipettors, thus avoiding gel excision and cDNA extraction procedures.


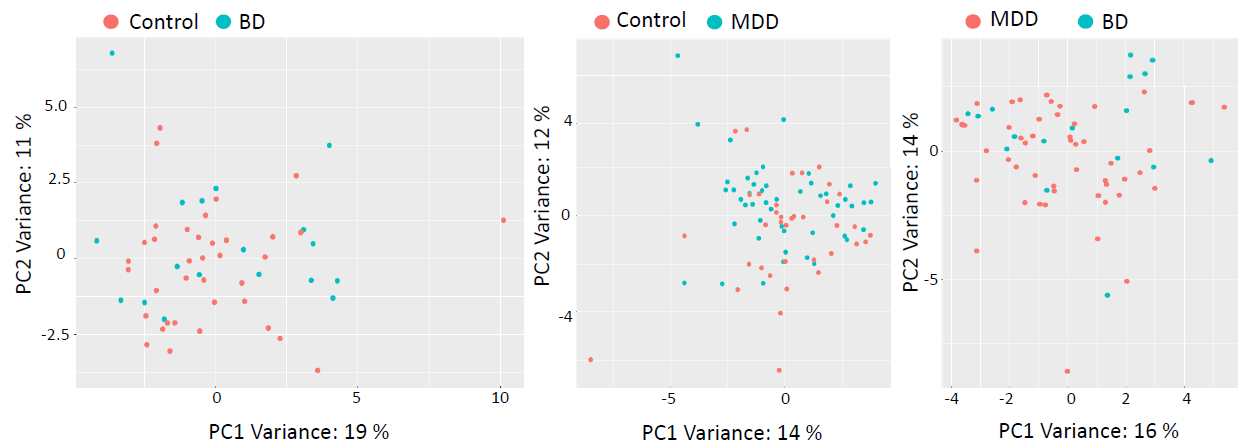


**Supplementary Figure 4.** The principal components analysis of the sample groups. Low circulating miRNA expression variance has been observed within sample groups.


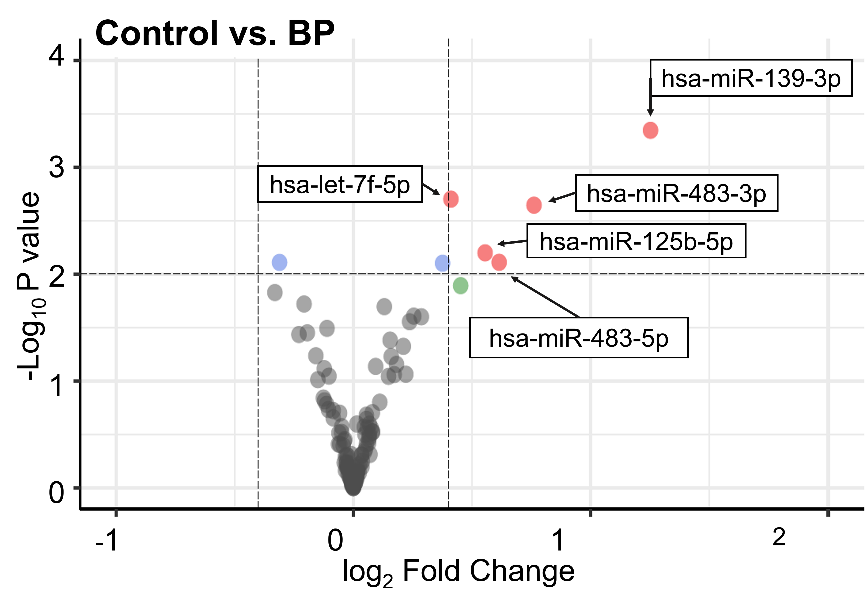


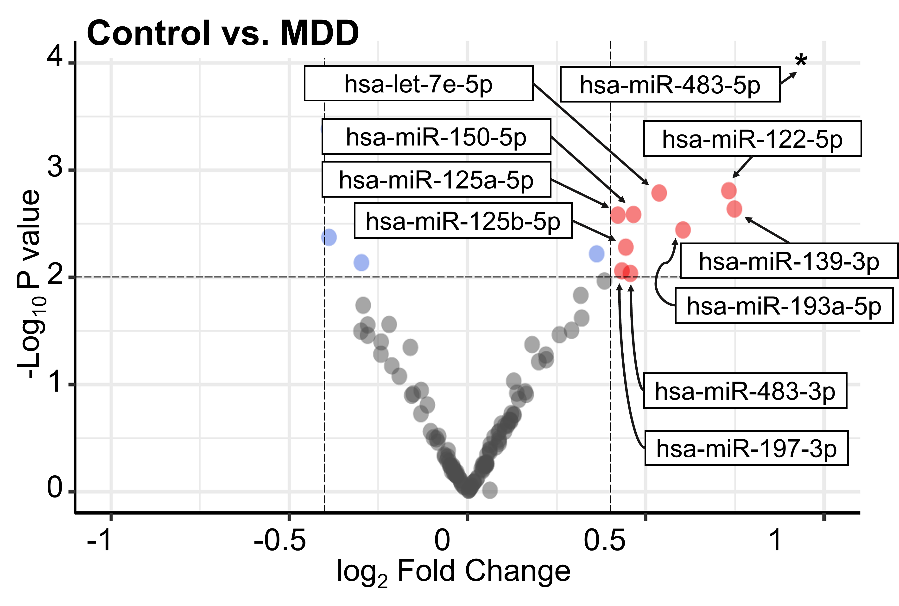


**
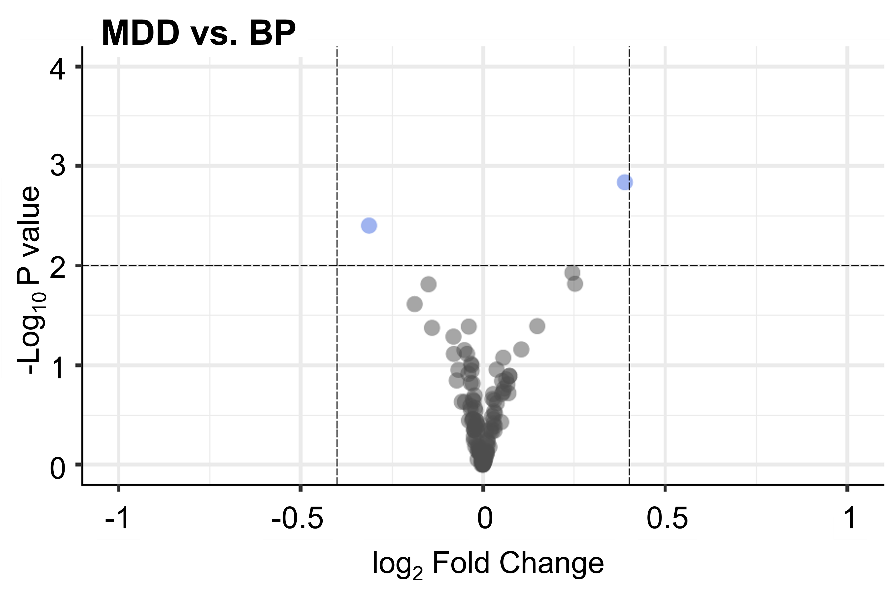
**

**Supplementary Figure 5.** Volcano plot of normalized differential miRNA expression NGS data in BD and MDD groups. * indicates Log2 Fold Change of 1.01 and p value <0.0001.
